# Linking data across multiple sectors to reduce health inequities among justice-involved young people in Australia

**DOI:** 10.23889/ijpds.v9i5.2568

**Published:** 2024-09-18

**Authors:** Lindsay Pearce, Lucas Calais-Ferreira, Rohan Borschmann, Matthew Legge, Susan Sawyer, Stuart Kinner

**Affiliations:** 1 Justice Health Group, School of Population Health, Curtin University; 2 Murdoch Children’s Research Institute and Royal Children’s Hospital, Melbourne; 3 School of Population and Global Health, University of Melbourne; 4 Department of Psychiatry, University of Oxford; 5 Department of Paediatrics, The University of Melbourne

## Background

A scarcity of evidence on the health and social needs of justice-involved young people contributes to persistent health and social inequities. Linking data from multiple sectors provides an opportunity to improve the health, justice, and social systems that this group interacts with, with a view to reducing these inequities.

## Methods

We established two population-level cohorts of justice-involved young people using multi-sectoral data linkage: (1) we linked youth justice records in Queensland from 1993-2014 for 48,670 young people to adult correctional records, death records, and coronial records; (2) we linked all youth justice records in Australia from 2000-2019 for 88,110 young people with national emergency department, hospital, primary care, pharmaceutical, and death records. We calculated mortality rates among young people exposed to the youth justice system and compared them with the age- and sex-matched general population.

## Results

Justice-involved young people in Queensland died at a 4.1 times higher rate than their non-justice-involved peers. Approximately one-third (34.6%) of deaths were from suicide. There was a 67% increased rate of death due to non-communicable diseases compared to the general population.

## Conclusions

The national cohort will fill evidence gaps on health needs and health care use after justice system contact. These two cohorts provide the first comprehensive look into the health of justice-involved young people in Australia and internationally. They form the foundation for routine monitoring and reporting on health needs and trajectories. Multi-sectoral data linkage is essential for sustainable, timely, and evidence-informed decision-making to improve systems, health, and mortality outcomes.

